# Development of Indirect Health Data Linkage on Health Product Use and Care Trajectories in France: Systematic Review

**DOI:** 10.2196/41048

**Published:** 2023-05-18

**Authors:** Florence Ranchon, Sébastien Chanoine, Sophie Lambert-Lacroix, Jean-Luc Bosson, Alexandre Moreau-Gaudry, Pierrick Bedouch

**Affiliations:** 1 Hospices Civils de Lyon Groupement Hospitalier Sud Unité de pharmacie clinique oncologique Pierre-Bénite France; 2 Translational Innovation in Medicine and Complexity - Unité Mixte de Recherche 5525 Université Grenoble Alpes Grenoble France; 3 Pôle Pharmacie Centre hospitalo-Universitaire Grenoble Alpes Grenoble France; 4 Faculté de Pharmacie Université Grenoble Alpes Grenoble France

**Keywords:** data linkage, health database, deterministic approach, probabilistic approach, health products, public health activity, health data, linkage, France, big data, usability, integration, care trajectories

## Abstract

**Background:**

European national disparities in the integration of data linkage (ie, being able to match patient data between databases) into routine public health activities were recently highlighted. In France, the claims database covers almost the whole population from birth to death, offering a great research potential for data linkage. As the use of a common unique identifier to directly link personal data is often limited, linkage with a set of indirect key identifiers has been developed, which is associated with the linkage quality challenge to minimize errors in linked data.

**Objective:**

The aim of this systematic review is to analyze the type and quality of research publications on indirect data linkage on health product use and care trajectories in France.

**Methods:**

A comprehensive search for all papers published in PubMed/Medline and Embase databases up to December 31, 2022, involving linked French database focusing on health products use or care trajectories was realized. Only studies based on the use of indirect identifiers were included (ie, without a unique personal identifier available to easily link the databases). A descriptive analysis of data linkage with quality indicators and adherence to the Bohensky framework for evaluating data linkage studies was also realized.

**Results:**

In total, 16 papers were selected. Data linkage was performed at the national level in 7 (43.8%) cases or at the local level in 9 (56.2%) studies. The number of patients included in the different databases and resulting from data linkage varied greatly, respectively, from 713 to 75,000 patients and from 210 to 31,000 linked patients. The diseases studied were mainly chronic diseases and infections. The objectives of the data linkage were multiple: to estimate the risk of adverse drug reactions (ADRs; n=6, 37.5%), to reconstruct the patient’s care trajectory (n=5, 31.3%), to describe therapeutic uses (n=2, 12.5%), to evaluate the benefits of treatments (n=2, 12.5%), and to evaluate treatment adherence (n=1, 6.3%). Registries are the most frequently linked databases with French claims data. No studies have looked at linking with a hospital data warehouse, a clinical trial database, or patient self-reported databases. The linkage approach was deterministic in 7 (43.8%) studies, probabilistic in 4 (25.0%) studies, and not specified in 5 (31.3%) studies. The linkage rate was mainly from 80% to 90% (reported in 11/15, 73.3%, studies). Adherence to the Bohensky framework for evaluating data linkage studies showed that the description of the source databases for the linkage was always performed but that the completion rate and accuracy of the variables to be linked were not systematically described.

**Conclusions:**

This review highlights the growing interest in health data linkage in France. Nevertheless, regulatory, technical, and human constraints remain major obstacles to their deployment. The volume, variety, and validity of the data represent a real challenge, and advanced expertise and skills in statistical analysis and artificial intelligence are required to treat these big data.

## Introduction

Data linkage is a technique for linking data from different sources that relate to the same person [1]. It increases the information available about each patient (clinical and administrative data, disease-related mortality, health care use, etc) and therefore expands research opportunities, particularly for research requiring large sample sizes, detailed data on hard-to-reach populations, or little loss to follow-up, to generate evidence with a high level of external validity [2,3]. The proliferation of the use of data linkage is reflected in the establishment of data linkage research centers worldwide [4] (Australia [5-7], North America [8,9], the Netherlands [10], the United Kingdom [11]). In Europe, a recent review highlighted national disparities in the integration of data linkage into routine public health activities [12].

In France, since the 2016 law on the modernization of the health system, the use of existing health data and their pooling have been promoted. As a result, French medicoadministrative databases (MADs) were linked together in a single French national health data system (*the Système National des Données de Santé* [SNDS]) in 2017 based on a reliable identification of individuals by the *numéro d’inscription au repertoire* (NIR), de-identified using 2 successive hash scrambling operations [13,14]. The French national health data system groups together (1) data on claims for reimbursement of outpatient care (the *Système National d’Information Inter-Régimes de l’Assurance Maladie* [SNIIRAM]), (2) medicoadministrative data on hospitalization in public and private hospitals (the *Programme de Médicalisation des Systèmes d’Information* [PMSI]), (3) the national death registry (the *Center d’Epidémiologie sur les causes médicales de Décès* [CépiDc]), and (4) medicosocial data (the *Caisse Nationale de Solidarité pour l’Autonomie* [CNSA]) [13-15]. The SNDS covers almost 99% of the French population, making it 1 of the largest databases of continuous homogeneous claims in the world [14]. Between 2007 and 2016, more than 400 scientific publications based on national health insurance data were identified in Medline, mainly on the real-life evaluation of drugs [13]. The consumption of reimbursed health products (ie, drugs and medical devices) for outpatients is recorded in the SNDS but not treatments used in hospitalized patients (except for expensive reimbursed drugs). Moreover, limited clinical data (no diagnosis, except in the case of hospitalization or chronic disease), no biological test results, scarce sociodemographic data, and no information about occupation [14,16] highlighted the potential for further linkage with other databases.

Clinical cohorts, disease-specific or population-based registries, and hospital data warehouses represent other databases of interest for data linkage in which reimbursed or nonreimbursed health products used in real life can be recorded, often in a heterogeneous way, depending on the initial purpose of the database. All these databases represent important tools for providing information about the safety and benefits of approved health products, with data on real-life use, rare outcomes, and long-term effects that were undetectable in randomized controlled trials [17]. Registries are particularly interesting for rare diseases, with more than 600 registries in Europe, for example, allowing development of clinical research in the field of rare diseases and so patient care improvement [18]. As these databases potentially represent different points in patient management, linking them can also make possible the reconstruction of care trajectories to better understand therapeutic management throughout the patient’s life.

Data linkage involves being able to match patient data, sometimes anonymized, between databases. The use of a common unique identifier to directly link personal data is often limited because it does not exist, is not regulatory available, or contains errors [19]. The national individual identifier “NIR” in French MADs is highly protected by privacy rules, leading to complex and lengthy formalities, which discourage potential applicants from using it [13,14,20,21]. Linkage with a set of indirect key identifiers has therefore been developed with deterministic and probabilistic approaches [2,4,22]. Deterministic linkage is applied when there are several identifiers that match perfectly between data sets. In this case, the match of a given identifier is evaluated as a discrete “all-or-nothing” outcome [23]. Probabilistic linkage uses statistical theory to associate each pattern of matching variable agreement with the likelihood that record pairs exhibiting the pattern are a match [19]. These data linkages are associated with a methodological challenge on linkage quality to minimize errors in linked data. Data linkage has to balance the risk of missed matches (failing to link data from the same individual) with false matches (mistakenly linking data from different individuals) [2]. As quality assessment of data linkage is essential to limit biased results and false interpretation, several recommendations highlight elements or information about the linkage pathway (data provision, method of data linkage, and data analyses) to be shared and checked [4,22]. In this context, the objective of this systematic review is to analyze the type and quality of research publications on indirect data linkage on health product use and care trajectories in France.

## Methods

### Search Strategy

A comprehensive search was performed by 2 authors (FR and SC) according to PRISMA (Preferred Reporting Items for Systematic Reviews and Meta-Analyses) guidelines (Multimedia Appendix 1) [24] for all papers published up to December 31, 2022, in 2 databases (PubMed/Medline and Embase databases). The search was conducted using a combination of various keywords from 3 categories. The first category included keywords related to different databases potentially used: SNDS, SNIIRAM, PMSI, CépiDc, electronic health record (EHR; Medical Subject Headings [MeSH] term), clinical data warehouse, claims database, registry (MeSH term), cohorts, and analyses (MeSH term). The second category of keywords was related to linkage: data linkage (MeSH term) and medical record linkage (MeSH term). The last category focused on the localization of the study and included 1 keyword: France (MeSH term). Keywords were organized using the following approaches: (1) keywords within 1 category were lined using the OR operator (eg, *SNDS* OR *PMSI*), and (2) keywords across different categories were connected using the AND operator (eg, *SNDS* AND *data linkage*).

### Study Selection

To be eligible for inclusion, studies had to be published in English or French, be human studies, be peer-reviewed papers, focus on health products (drugs or medical devices) or care trajectories, and involve the use of linked French data defined as the linking of 2 or more French data collections at the patient level (Table 1).

Only studies based on the use of indirect identifiers were included (ie, without a unique personal identifier available to easily link the databases—the NIR for French databases). Studies on data linkage between the French MADs making up the SNDS were not identified (eg, between SNIIRAM and the PMSI). A manual review of the reference lists of all selected papers was performed to identify any other relevant studies. A full-text search was performed when it was not possible to determine from the abstract whether linked data were used.

**Table 1:** Inclusion and exclusion criteria for the selection of papers for the review.

Criteria	Included	Excluded
Paper type	Primary research studies	Editorials, letters, commentaries, book chapters, studies where the full text of the publication was not available
Intervention	Indirect data linkage	Direct data linkage (ie, unique personal identifier available to easily link the databases—the NIR^a^ for French databases); studies on data linkage between the French MADs^b^ making up the SNDS^c^ not identified (eg, between SNIIRAM^d^ and the PMSI^e^)
Outcome	Studies on health product (drugs or medical devices) use or care trajectories of patients	N/A^f^
Language	English, French	Non-English
Time frame	Up to December 31, 2022	N/A

^a^NIR: numéro d’inscription au repertoire.

^b^MAD: medicoadministrative database.

^c^SNDS: Système National des Données de Santé.

^d^SNIIRAM: Système National d’Information Inter-Régimes de l’Assurance Maladie.

^e^PMSI: Programme de Médicalisation des Systèmes d’Information.

^f^N/A: not applicable.

### Data Extraction and Analysis

The full text of all the eligible papers was screened, and the following criteria were collected: date of publication, research area (type of health products, care trajectories), type of database linked (MAD, registry [set of disease events occurring in a well-characterized population over a given period of time] [25], cohort [group of individuals sharing a certain number of features and who are followed longitudinally, at the individual level, according to a pre-established protocol] [26], other databases), number of patients in the different databases, and linkage method (deterministic, probabilistic, or alternative approaches) [27]. Data linkage quality was assessed using analysis quality indicators and adherence to the Bohensky framework for evaluating data linkage studies [4] on selected studies published since 2011 (ie, the date of publication of this guide). The Bohensky tool is a checklist for reviewing the specific sources of bias in interpreting the findings of clinical research studies based on data linkage, including the data sets being used, the linkage variables and process, and an assessment of the quality of the linked data sets [4].

## Results

### Literature Search Findings

**Figure 1:**
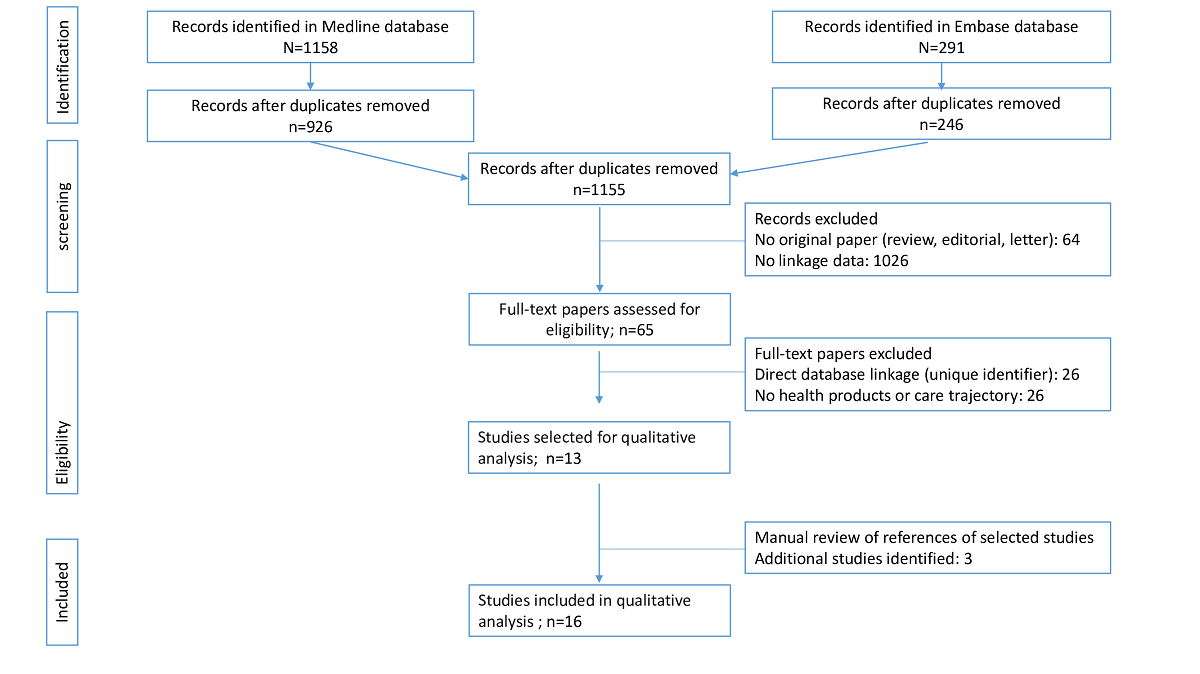
Flowchart of study selection.

The systematic literature search identified 1155 papers. Screening against the inclusion and exclusion criteria retrieved 39 (3.4%) papers using health database linkage, of which 16 (41.0%) focused on health care product use and care trajectories for the final analysis (Figure 1). Most papers were recent, with over 87% (n=14) of these papers published since 2017 (Multimedia Appendix 2 [27-42]). In 1 (6.3%) case included in this review, the publication corresponded to a study protocol in which the data linkage was planned with varying degrees of accuracy [28]. Data linkage was performed at the national level (ie, for all patients with a disease or who received therapy in France) in 7 (43.8%) cases [27-33] or at the local level in 9 (56.2%) studies [34-42]. The number of patients included in the different databases (medicoadministrative, registries, cohorts, etc) varied greatly, ranging from 713 patients [34] to 75,000 patients [39,41]. The number of patients resulting from data linkage was also variable, ranging from 210 patients [34] to 31,000 linked patients [27].

### Research Areas

The health products covered by the studies included drugs (anticoagulants or antiplatelet agents [34,36-38], 5-alpha-reductase inhibitors [39,41], immunosuppressive treatments [32,33], antihypertensive drugs [31,34], and statins [34]) and medical devices (transcatheter aortic valve implantation [27]). The diseases studied were mainly chronic diseases (chronic kidney disease [28-33,35]; cancer [35,39,41]; cardiovascular diseases, eg, arterial hypertension [40], aortic stenosis [27], stroke [34]; diabetes [40]) and infections [33].

**Figure 2:**
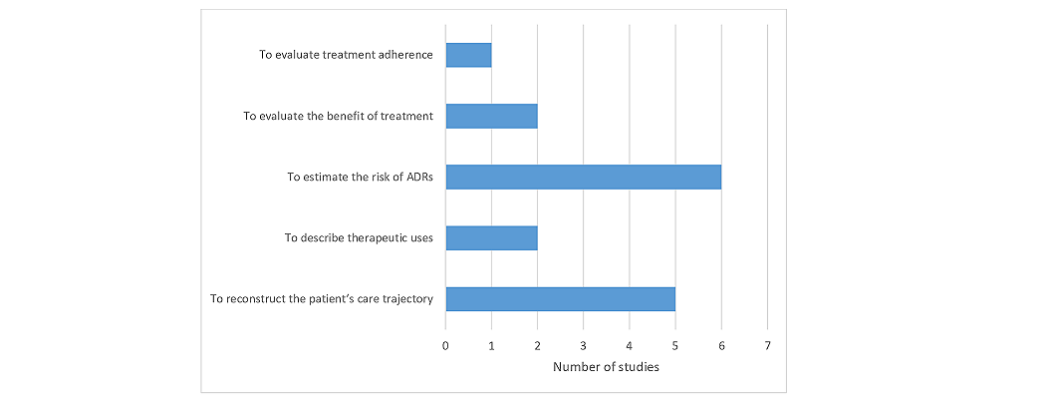
Objectives of studies using data linkage. ADR: adverse drug reaction.

Although data linkage increases the information available on each patient, the objectives of the data linkage were multiples: to estimate the risk of adverse drug reactions (ADRs) (n=6, 37.5%) [33,36-39,41], to reconstruct the patient’s care trajectory (n=5, 31.3%) [28-30,40,42], to describe therapeutic uses (n=2, 12.5%) [32,35], to evaluate the benefits of treatments (n=2, 12.5%) [27,31], and to evaluate treatment adherence (n=1, 6.3%; see Figure 2) [34].

### Type of Database Linkage

All (n=15, 93.7%) but 1 (6.3%) study [35] reported indirect linkage using French claims databases (SNIIRAM, the PMSI, or the SNDS) at the national or the regional level. Registries are the most frequently linked databases with French claims data, with 6 (37.5%) studies based on the Renal Epidemiology and Information Network (REIN) registry [28-33]. The REIN registry records information about all patients with end-stage renal disease who start renal replacement therapy (dialysis or pre-emptive kidney transplantation) in France [43]. It includes data on patient and center identification, primary kidney disease, initial clinical characteristics, comorbidities, and management of end-stage renal disease, but it does not contain data on health care consumption. In addition, 2 (12.5%) studies involved registries of cardiological procedures [27,42], and 1 (6.3%) study involved cancer registries [35].

Regarding cohort databases, Mechtouff et al [34] linked the AVC69 cohort (patients with suspected acute stroke admitted to an emergency department or stroke unit in the Rhône area) and a MAD to assess the use of and adherence to secondary prevention drugs 3 and 6 years after a transient ischemic attack or ischemic stroke. Ad hoc emergency hospital clinical databases from 5 regions in France (Angers, Brest, Grenoble, Nantes, and Rennes) were used to study the risk of major bleeding with anticoagulant and antiplatelet agents [36-38].

Finally, 3 (18.8%) studies focused on outpatient data. The results of biological explorations (Gleason score on prostate biopsy sample) from pathological anatomy laboratories in Brittany were linked with a MAD (SNIIRAM) in 2 (12.5%) studies in order to assess the risk of prostate cancer in patients treated with 5-alpha-reductase inhibitors for symptomatic benign prostatic hyperplasia [39,41]. Perlbarg et al [40] reported on the feasibility of matching a general practice database from ambulatory care with the French health insurance database (SNIIRAM), which represents a novel approach to analyze treatment, comorbidities, medical practices, and care pathways. No studies looked at linking with a hospital data warehouse, a clinical trial database, or patient self-reported databases.

### Linkage Method

In this review, the deterministic approach [28-31,39,41,42] was used in 7 (43.8%) studies and the probabilistic linkage in 4 (25.0%) studies [27,32,33,40]. In addition, 5 (31.3%) studies used linkage with indirect key identifiers but did not specify whether it was a deterministic or a probabilistic approach [34-38]. They all performed data linkage at the local level. The linkage rate, when specified in the study (11/15, 73.3%, studies), was mainly from 80% to 90% [27,29,31,39,41]. Only 3/15 (20%) studies achieved a rate of over 90% [30,33,42].

The number of key identifiers varied from 3 [34,35] to 11 [42]. The application or software used for the linkage was poorly described. For some studies, manual linkage was probably performed but not clearly described.

### Quality of the Linkage

As the publication of Raffray et al [28] was a study protocol in which the results of data linkage were not specified, the quality of the data linkage was not assessed.

Analysis of the characteristics of matched versus unmatched records to assess the completeness of the linkage or to identify a potential selection bias (ie, a specific population might be missed by the algorithm) was used in 26.7% (4/15) of the studies [27,29,30,42]. Didier et al [27] used comparisons of survival curves between merged populations, registries, and the SNDS to assess the quality of the linkage results. Another method to determine whether a pair is a true match was to check that the comorbidity recorded in database 1 was also recorded in database 2 for all linked patients. This method was used by Raffray et al [29] to compare diabetes in matched patients. The sensitivity of linkage (ie, the proportion of truly matched records detected), specificity (ie, the proportion of truly unmatched records detected), and the positive predictive value and negative predictive value were reported in only 1 (6.7%) of 15 studies at 99.9%, 97.9%, 99.9%, and 96.9%, respectively [42].

Finally, adherence to the Bohensky framework for evaluating data linkage studies [4] is presented in Table 2. This analysis showed that the description of the source databases for the linkage was always performed but that the completion rate and accuracy of the variables to be linked were not systematically described.

**Table 2:** Adherence to the Bohensky framework.

Framework item and description			Studies																				
			Perlbarg et al [40]		Béchade et al [35]		Hogan et al [33]		Sitruk et al [32]		Mechtouff et al [34]		Scailteux et al [39]		Lesaine et al [42]		Ferrerira et al [31]		Bouget et al [36-38]		Didier et al [27]		Raffray et al [29,30]
**1. Completeness of source databases**																							
	A description of the data sources to be used in the study should be included.	Given		Given		Given		Given		Given		Given		Given		Given		Given		Given		Given	
	The number of eligible records obtained from each data set and the reasons for differences should be reported.	Given		Given		Given		Not performed		Given		Given		Given		Not performed		Given		Given		Given	
**2. Accuracy of data sources**																							
	Variables selected by researchers for linkage and analysis should be reported.	Given		Given		Given		Given		Given		Given		Given		Given		Given		Given		Given	
	The completion rate and accuracy of variables to be linked should be presented.	Given		Not performed		Not performed		Not performed		Not performed		Not performed		Given		Not performed		Given		Given		Given	
	Coding practices and the use of standardized definitions should be stated, if used.	Given		Given		Given		Given		Given		Given		Given		Given		Given		Given		Given	
**3.** **Linkage methodology and technology**																							
	A measure of the validity of the linked data sets (false-positive and false-negative rates, if available) should be given.	Not performed		Not performed		Not performed		Not performed		Not performed		Not performed		Given		Not performed		Not performed		Given		Given	
	An analysis of potential sources of bias among nonlinked cases should be reported.	Given		Not performed		Not performed		Not performed		Not performed		Given		Given		Not performed		Given		Given		Given	
	The denominator used to derive linkage rates and the justification for this should be reported.	Given		Not performed		Not performed		Not performed		Not performed		Given		Given		Not performed		Not performed		Given		Given	
	A description of the data linkage methods (ie, deterministic or probabilistic) with a justification for these should be reported.	Given		Not performed		Given		Given		Not performed		Given		Given		Given		Not performed		Given		Given	

## Discussion

### Principal Findings

Health data linkage is a powerful research resource that is being increasingly developing worldwide but with national disparities [12,44]. This review aimed to share research publications on indirect health data linkage focusing on health product use and care trajectories in a country with a centralized health data system. As the French claims database is 1 of the largest in the world, covering almost the whole population (99%) from birth to death, irrespective of provider, socioeconomic status, or retirement [14], linkage with other data offers great research potential to study the safety and effectiveness of drugs in routine care [16]. Nevertheless, only 16 studies describing their indirect record linkage methodology were included in our review. They were mainly published since 2017, which corresponds to the start of facilitated access to health data, which was also illustrated with the increase in accepted health data access projects after the reform [20]. This result suggests that the recent increase in data linkage in France is encouraging and needs to be confirmed. This review also highlights that indirect linkage mainly actually involves claims databases and disease-specific or population-based registries. However, many others database, including the use of health products, exist, emphasizing the big potential and challenges associated with data linkage. Finally, the quality of data linkage is poorly described: few studies have assessed or specified potential errors associated with data processing before, during, and after linkage. Moreover, 31% of the studies included in this review did not define the method of indirect data linkage (ie, deterministic or probabilistic approaches). Wider appropriation and dissemination of recommendations for the proper use of database linkage seem important to achieve.

Comparing our results on the use of health data linkage to other countries is difficult due to the use of different linkage models [2,12]. For example, Young et al [1] reported over 1200 publications in Australia based on linked data since 2009, without specifying the linkage method. In 2020, Haneef et al [12] reported in their European survey that France uses advanced data linkage for routine public health activities at national and subnational levels. As direct linkage between French health databases involves the use of a unique identification number (NIR), and because its sharing is highly protected, we focused in this review on indirect linkage. However, several large health cohorts have been directly linked to the SNDS [26]^.^ For example, CONSTANCES is a large prospective population-based cohort (200,000 persons included), in which an annual direct linkage is performed with 3 French social and health databases (SNIIRAM; Caisse nationale d'assurance vieillesse [CNAV], the national salaried employees retirement fund; and the National Death Registry, CépiDc) [45-47]. Another example of a database directly linked to the SNDS, and not included in its formal framework, is Resid’EPHAD, which allows nursing homes to transmit information about the residents and their health care consumption [13,48].

Claims databases and disease-specific or population-based registries appeared to be the most used database for data linkage on health product use and care trajectories in this review. Nevertheless, other databases are likely to be linked in the future. Health insurance claims databases and EHRs have been identified as the preferred data sources for studying the safety and effectiveness of drugs in routine care [49]. Surprisingly, Lin and Schneeweiss [49] reported in 2016 in their review only 9 papers on linking electronic medical records to claims data to study drug safety and effectiveness. All the included studies were found to be based in the United States, although this was not part of the inclusion criteria [49]. The development of hospital data warehouses, defined as a large computerized database that processes all data generated during hospital stays from the hospital information system (eg, medical observations and diagnoses, biology, imaging, prescribing, and drug administration), is on the rise [15,50-52]. These databases can quickly become powerful tools because real-time data collection is automated and reflects the clinical practice in hospitals for all inpatients [15]. In particular, they offer the possibility of supplementing data on health products used in hospitalized patients, which are data rarely captured in other databases [44]. The pharmaceutical record (the *Dossier Pharmaceutique*), which is a centralized national electronic database shared by all French community pharmacists on all dispensing, could provide added value to study over-the-counter drug consumption [53,54]. Another possible source of data to be linked to health databases is data generated by new tools, from various fields, such as mobile phones, social networks, eHealth, and connected medical devices. The quantified self-tracking movement, defined as the regular collection of any measurable data about oneself, such as biological, physical, behavioral, or environmental information, offers an additional opportunity to enrich knowledge about health product use and care trajectories [55]. Patient-reported outcomes are increasingly used in routine medical care, improving patient-clinician communication, clinician knowledge of symptoms, symptom management, patient satisfaction, quality of life, and overall survival [56]. In this context, Tran et al [57] proposed the “COOP’ e-cohort,” which aims to build a large community of patients willing to participate in research by contributing to the creation of a large database, passively enriched, at the individual level, through linkage with routinely collected care or medicoadministrative data. All of these real-world data offer the possibility of describing new insights into the use of health products in daily clinical practice [58].

In this review, we chose the Bohensky framework for evaluating data linkage studies [4] because of its simplicity of use. Nevertheless, more comprehensive recent guidelines (Guidance for Information about Linking Data sets [GUILD] guidance) [22] and a checklist (expanded Reporting of Studies Conducted Using Observational Routinely Collected Health Data for Pharmacoepidemiology [RECORD-PE]) [59] have been developed on the information that needs to be made available about the data linkage process by data providers, data linkers, analysts, and researchers and could have provided further relevant information. The methodology chosen for record linkage has the potential to introduce misclassification into research studies and should be discussed [60]. Efforts need to be made to improve the health scientific community’s understanding of data linkage methodology and the interpretation of linked data. The recent creation of the Health Data Hub [61], enabled by the law of July 24, 2019, on the organization and transformation of the French health system [21] should improve this. Its objective is to enable the implementation of authorized innovative projects using nonnominal data via a state-of-the-art secure technological platform [21]. Once all the regulatory authorizations relating to data security have been obtained, the Health Data Hub platform would centralize databases from patient registries, cohorts, or electronic medical records and allow the linkage of consolidated databases with SNDS data [21].

### Limitations

Limitations of this review include our research strategy based on the MeSH terms “medical record linkage” and “data linkage,” which probably excluded some potentially eligible studies that were not referenced with these terms. In addition, Tuppin et al [13] highlighted a lack of homogeneity in the English terms used to describe French national health insurance databases, which made it more difficult to detect papers. Therefore, the use of indirect data linkage may be underestimated in our review. Moreover, some interesting information on data linkage studies may have escaped a traditional bibliography (ie, reports provided to health authorities or other gray literature) and may thus contribute to the underreporting of indirect data linkage experiences in France. Wider application of RECORD guidelines [62], which aims to increase the discovery of publications involving the use of routinely collected data, including data linkage, would help overcome this underreporting issue.

### Implications for the Future

For French health organizations and regulators, this review suggests that efforts initiated with the creation of the Health Data Hub be continued in order to facilitate the reuse of data, while ensuring respect of data privacy. A European dimension is also planned for sharing with other databases. One of the points raised in this review is also the need to promote and intensify the training in health data science in medical and pharmaceutical universities in collaboration with data scientists.

### Conclusion

This review highlighted the growing interest in health data linkage on health product use and care trajectories in France. Nevertheless, regulatory, technical, and human constraints remain major obstacles to their deployment. The volume, variety, and validity of the data represent a real challenge, and advanced expertise and skills in statistical analysis and artificial intelligence are required to treat these big data.

## Appendix

Multimedia Appendix 1 PRISMA (Preferred Reporting Items for Systematic Reviews and Meta-Analyses) guidelines.

Multimedia Appendix 2 Description of included studies (N=16).

## Abbreviations

ADR: adverse drug reaction
CépiDc: Center d’Epidémiologie sur les causes médicales de Décès
EHR: electronic health record
MAD: medicoadministrative database
MeSH: Medical Subject Headings
NIR: numéro d’inscription au repertoire
PMSI: Programme de Médicalisation des Systèmes d’Information
REIN: Renal Epidemiology and Information Network
SNDS: Système National des Données de Santé
SNIIRAM: Système National d’Information Inter-Régimes de l’Assurance Maladie
