# Supplementary material for: Development of Indirect Health Data Linkage on Health Product Use and Care Trajectories in France: Systematic Review
Source: J Med Internet Res. 2023 May 18;25:e41048. doi: 10.2196/41048 (PMC10236279; doi:10.2196/41048)
Supplement: Multimedia Appendix 2 [file jmir_v25i1e41048_app2.docx]

**Multimedia Appendix 2.** Description of included studies (N=16).

| Author | Database linkage | Research areas | Objective of linkage | Database 1 | Patients in database 1, N | Database 2 | Patients in database 2, N | Linkage method | Linkage identifiers | Linked patients, n | Percentage matching (%) | Level |
| --- | --- | --- | --- | --- | --- | --- | --- | --- | --- | --- | --- | --- |
| Perlbarg et al [40] | MAD^a^: General Practice Observatory database | Diabetes/arterial hypertension | To reconstitute the patient’s care pathway | SNIIRAM^b^ | 35,730 | General Practice Observatory database | 37,992 | Probabilistic | National doctor number, date of meeting, type of meeting (consultation, home visit), month and year of birth, sex | 29,088 | 76.5 | Local |
| Béchade et al [35] | Registry: registry and hospital database | Cancer/chronic kidney disease | To assess long-term patient follow-up | FRANCIM^c^ | 44,354 | Hospital database and National Peritoneal Dialysis registry | 2881 | Not detailed | Family name, first name, date of birth | 234 | Not detailed | Local |
| Hogan et al [33] | MAD: registry | Chronic kidney disease/infection | To estimate ADRs^d^ | Renal transplant database | 680 | PMSI^e^ | 632 | Probabilistic | Age, sex, month and year of transplantation, district of treatment | 593 | 94 | National |
| Sitruk et al [32] | MAD: registry | Chronic kidney disease/treatment | To describe the therapeutic strategy | SNDS^f^ | 2463 | Renal transplant database CRISTAL: REIN^g^ | 2552 | Probabilistic | Sex, age, residence postcode, center identification number, month and year of renal transplantation | 1598 | 65 | National |
| Mechtouff et al [34] | MAD: cohort | Stroke | To assess long-term patient follow-up: adherence | Rhône-Alpes ERASME^h^ | Not detailed | AVC69 cohort (patients admitted to an emergency or stroke unit in the Rhône area) | 713 | Not detailed | Name, first name, date of birth | 210 | Not detailed | Local |
| Scailteux et al [39] | MAD: laboratory database | Cancer/ADRs | To estimate ADRs | SNIIRAM | (a) 74,596  (b) 1055 | Pathology laboratory database | 13,796 prostate results | Deterministic | Month and year of men’s birthdays, date (month day year) of prostate sampling in SNIIRAM, date of prostate sample reception in pathology laboratory database (or date of sampling in case of date missing) | 859 | 81.4 | Local |
| Ferreira et al [31] | MAD: registry | Chronic kidney disease | To estimate benefit of treatments | SNDS | Not detailed | REIN registry | 17,046 | Deterministic | Sex, age, residence postcode, RRT^i^ center identification number, month and year of dialysis start, month and year of death | 14,698 | Not detailed | National |
| Raffray et al [28]^j^ | MAD: registry | Chronic kidney disease | To reconstitute the patient’s care pathway | SNDS | Not detailed | REIN registry | Not detailed | Deterministic | Sex, age, residence postcode, RRT center identification number, month and year of dialysis start, month and year of death | N/A^k^ | N/A | National |
| Scailteux et al [41] | MAD: laboratory database | Cancer/ADRs | To estimate ADRs | SNIIRAM | (a) 74,596  (b) 1055 | Pathology laboratory database | 13,796 prostate results | Deterministic | Month and year of men’s birthdays, date (month day year) of prostate sampling in SNIIRAM, date of prostate sample reception in pathology laboratory database (or date of sampling in case of date missing) | 859 | 81.4 | Local |
| Bouget et al [38] | MAD: ad hoc clinical database | ADRs | To estimate ADRs | SNIIRAM | 3,83,557 hospital stays | Clinical emergency database | 6354 events | Not detailed | Date of birth (month, year), gender, date (day, month, year) of hospital entry ±3 days, type of antithrombotic drug, geographic area of first antithrombotic drug delivery and care facilities | 5264 events | Not detailed | Local |
| Raffray et al [29] | MAD: registry | Chronic kidney disease | To reconstitute the patient’s care pathway | SNDS | 28,402 | REIN registry | 22,073 | Deterministic | Age, sex, residence, treatment center, treatment date, death date | 19,223 | 87.1 | National |
| Bouget et al [36] | MAD: ad hoc clinical database | ADRs | To estimate ADRs | SNIIRAM | 69,992 | Clinical emergency database | Not detailed | Not detailed | Date of birth (month, year), gender, date of hospital entry and discharge, type of antiplatelet drug, and care facility involved | 250 | Not detailed | Local |
| Bouget et al [37] | MAD: ad hoc clinical database | ADRs | To estimate ADRs | SNIIRAM | 47,469 | Clinical emergency database | Not detailed | Not detailed | Date of birth (month, year), gender, date of hospital entry and discharge, type of antiplatelet drug, care facility involved | 573 | Not detailed | Local |
| Didier et al [27] | MAD: registry | Aortic stenosis/TAVI^l^ | To assess long-term patient follow-up | SNDS | Not detailed | France-2 registry and France TAVI registry | 34,397 | Probabilistic | Primary linkage variable: CCAM^m^ code for procedure, sex, year of birth, hospital national code, date of discharge ±3 days  Secondary linkage variable: hospital admission ±3 days, same dates of hospital discharge, year and month of birth, CCAM code for procedure, sex, hospital national code | 30,913 | 89.9 | National |
| Raffray et al [30] | MAD: registry | Chronic kidney disease | To reconstitute the patient’s care pathway | SNDS | Not detailed | REIN registry | 10,667 | Deterministic | Sex, age, residence postcode at the time of the considered RRT event, RRT center identification number, month and year of the RRT event (renal transplant, first dialysis, or endpoint), month and year of patient’s death | 9627 | 90.3 | National |
| Lesaine et al [42] | MAD: registry | Coronary intervention | To reconstitute the patient’s care pathway | PMSI of Aquitaine Region | Not detailed | Interventional cardiology registry (ACIRA^n^) | 26,618 | Deterministic | Age, sex, month and year of hospital discharge, hospital code FINESS^o^, code of residency, principal diagnosis, related diagnosis, length of stay, number of associated diagnoses, homogeneous group of patients | 25,773 | 96.8 | Local |

^a^MAD: medicoadministrative database.

^b^SNIIRAM: Système National d’Information Inter-Régimes de l’Assurance Maladie, the reimbursement claims data for ambulatory care from the French health care insurance system.

^c^FRANCIM: French Network of Cancer Registries.

^d^ADR: adverse drug reaction.

^e^PMSI: Programme de Médicalisation des Systèmes d’Information, the medicoadministrative hospitalization data from public and private hospitals.

^f^SNDS: Système National des Données de Santé, the French MAD.

^g^REIN: Renal Epidemiology and Information Network.

^h^ERASME: Extraction, Recherches, Analyses pour un Suivi Medico-Economique, the medical reimbursement database of the French National Health Insurance.

^i^RRT: renal replacement therapy.

^j^This paper is a study protocol.

^k^N/A: not applicable.

^l^TAVI: transcatheter aortic valve implantation.

^m^CCAM: Common Classification of Medical Procedures.

^n^ACIRA: Registre Aquitain de Cardiologie Interventionnelle.

^o^FINESS: Fichier national des établissements sanitaires et sociaux.
